# Supplementary figures and images for: Dynamic trafficking and turnover of JAM-C is essential for endothelial cell migration
Source: PLoS Biol. 2019 Dec 2;17(12):e3000554. doi: 10.1371/journal.pbio.3000554 (PMC6907879; doi:10.1371/journal.pbio.3000554)

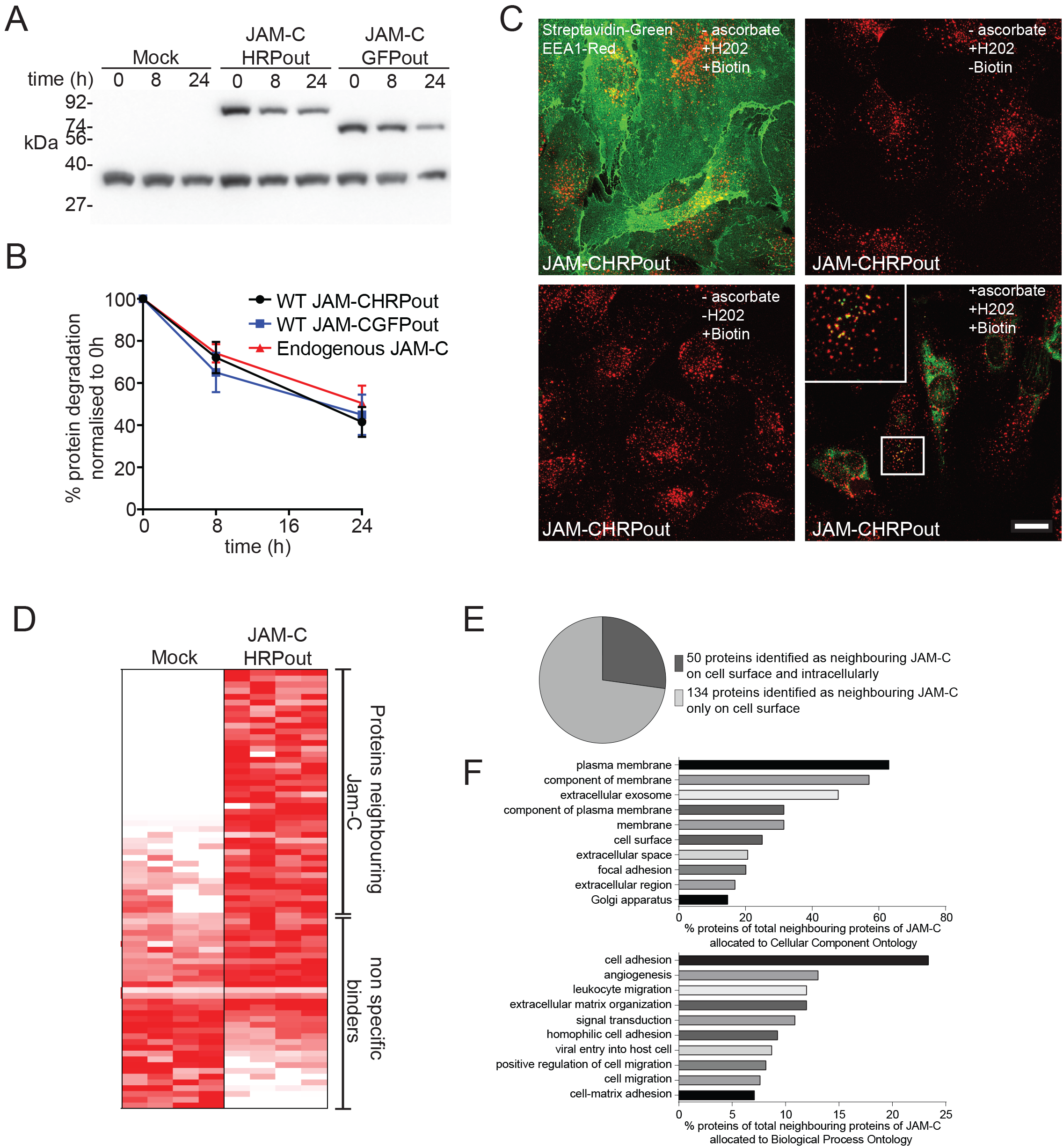

Supplement: S1 Fig — (A and B) HUVECs were transfected with WT JAM-C–HRPout or JAM-C–GFPout constructs and treated with 10 μg/ml cycloheximide. (A) The rate of protein degradation was determined by SDS-PAGE and western blot over a 24-h period, and a representative anti-JAM-C blot is shown. (B) Quantification of western blots normalised to the 0-h protein levels; data from n = 4 experiments, error bars represent SEM. Underlying data are found in S7 Data. (C–F) HUVECs were transfected with JAM-C–HRP and on the following day incubated with or without biotin tyramide and hydrogen peroxide in the presence or absence of 50 mM ascorbate. (C) Immunofluorescence analysis of cells labelled for streptavidin (green) and EEA 1 (red). Biotinylated proteins are only present when both hydrogen peroxide and biotin tyramide is present and partially localise with early endosomes. (D–F) Biotinylated proteins were pulled down with streptavidin and analysed by mass spectrometry (n = 4 experiments). (D) An example data set from one mass spectrometry experiment is shown, consisting of duplicate samples with each mass spectrometry run repeated. Proteins near JAM-C appear only in transfected cells. Proteins that appear solely in mock or in mock and JAM-C-HRP–transfected sample represent nonspecific binders. (E) Pie chart showing the number of proteins adjacent to JAM-C at the cell surface and intracellularly. (F) The percentage of protein hits associated with specific cellular locations and processes is plotted. EEA 1, early endosome antigen 1; GFP, green fluorescent protein; HRP, horseradish peroxidase; HUVEC, human umbilical vein endothelial cell; JAM-C, junctional adhesion molecule-C; SEM, standard error of the mean; WT, wild type. (TIF) [file pbio.3000554.s001.tif]

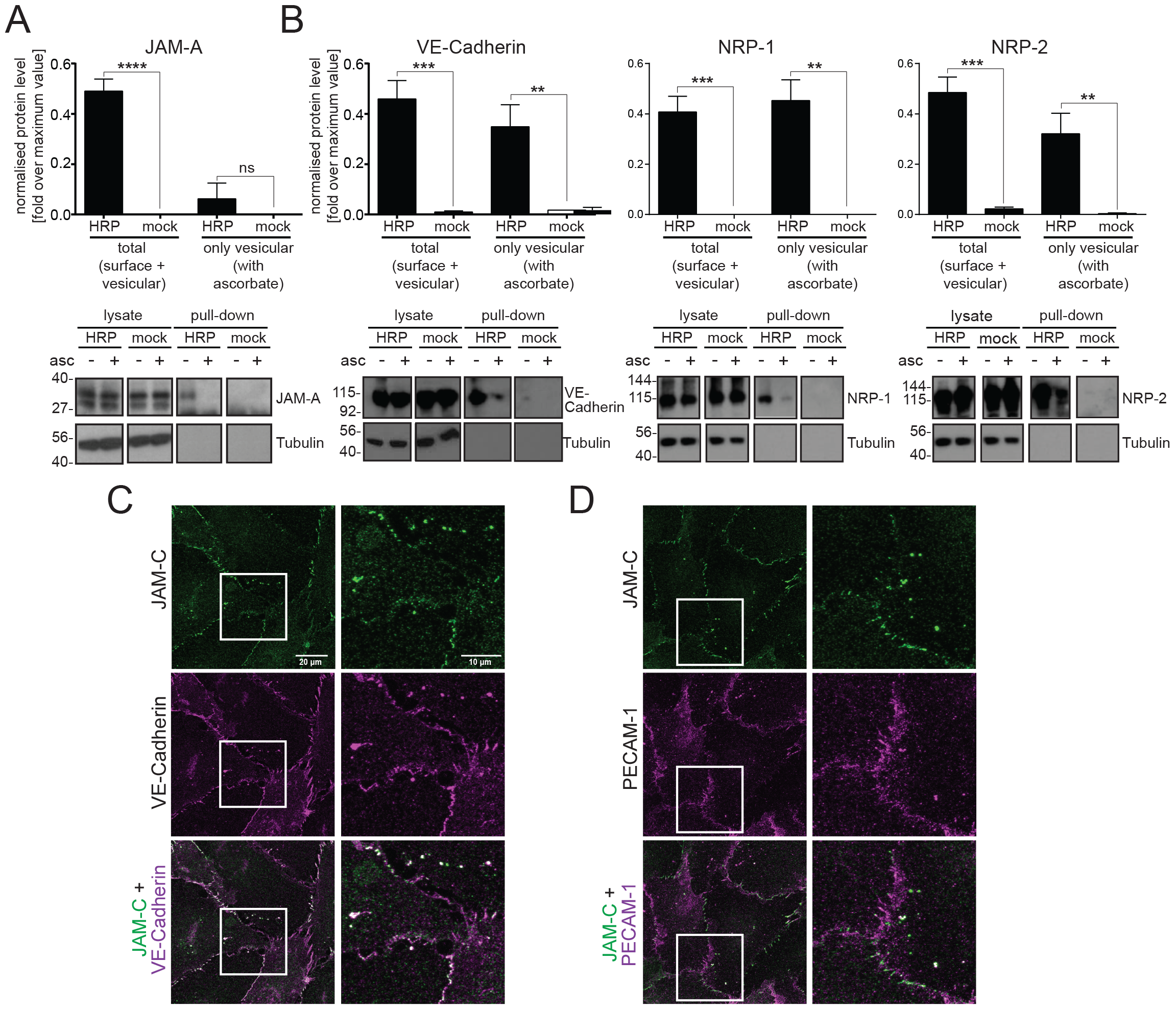

Supplement: S2 Fig — (A and B) JAM-C-HRPout–transfected cells were fed with biotin tyramide and exposed to hydrogen peroxide in the presence or absence of ascorbate. Biotinylated proteins were pulled down and western-blotted for (A) proteins neighbouring JAM-C at the cell surface: JAM-A or (B) proteins cotrafficked with JAM-C: VE-Cadherin, NRP-1, and NRP-2. Representative blots are shown with quantification of n = 4 experiments, and error bars represent SEM (*p ≤ 0.05, ** p ≤ 0.01; ***p ≤ 0.001, ****p ≤ 0.0001; unpaired t test). (C) Immunofluorescence analysis of endogenous JAM-C (green) and either VE-Cadherin or PECAM-1 (magenta). The boxed region is magnified. VE-Cadherin cotraffics with JAM-C, whilst PECAM-1 does not. Underlying data are found in S8 Data. HRP, horseradish peroxidase; JAM-C, junctional adhesion molecule-C; NRP, neuropilin; PECAM-1, platelet endothelial cell adhesion molecule 1; SEM, standard error of the mean; VE-Cadherin, vascular endothelial cadherin. (TIF) [file pbio.3000554.s002.tif]

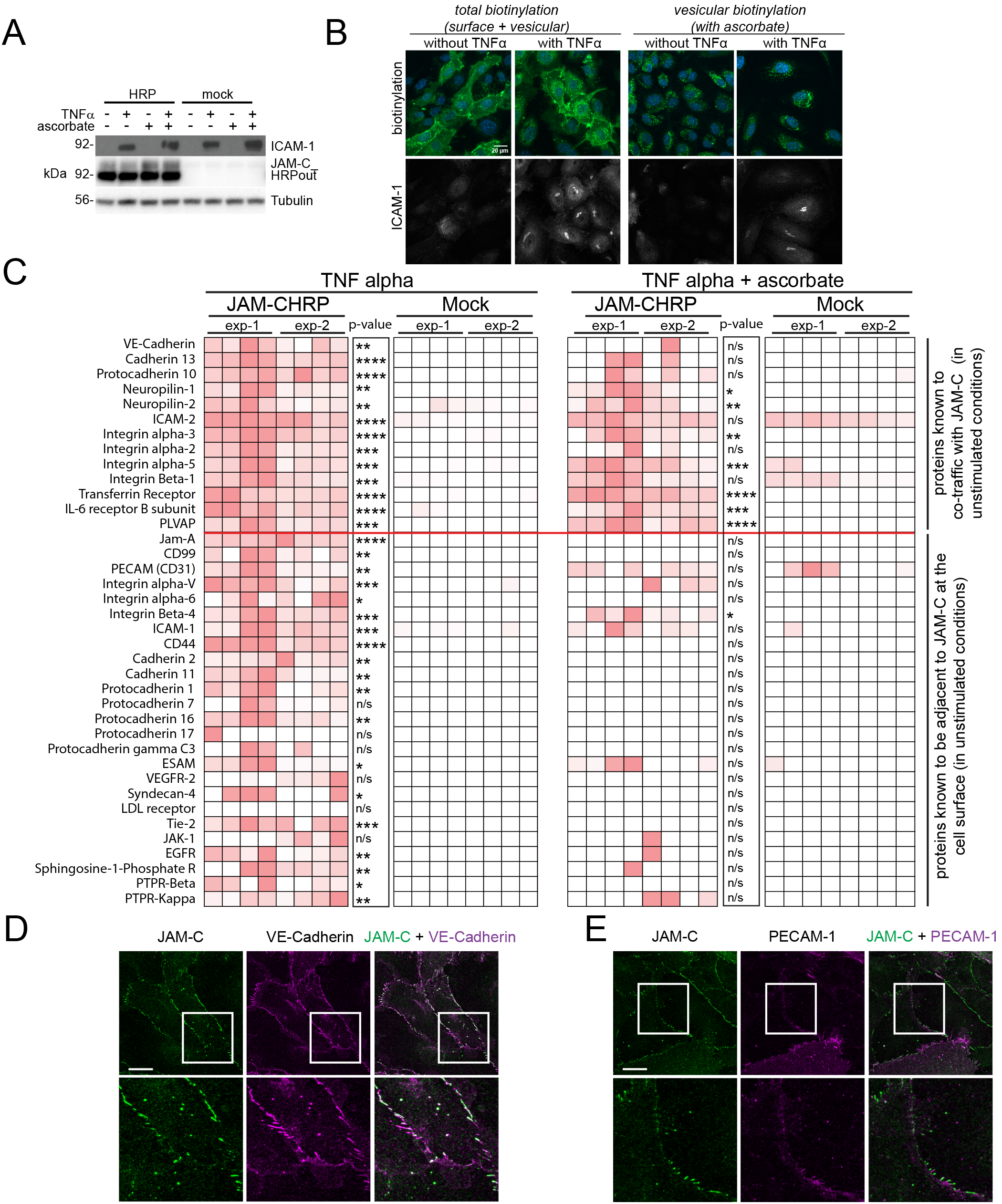

Supplement: S3 Fig — (A–C) HUVECs were transfected with JAM-C–HRPout and stimulated for 4 h with 50 ng/ml TNF-α. (A) Cells were lysed and analysed by western blot. The level of JAM-C–HRP expression is similar across all transfected samples, and TNF-α stimulation up-regulates the expression of ICAM-1. (B and C) Cells were fed biotin tyramide for 30 min and then exposed to hydrogen peroxide for 1 min in the presence or absence of 50 mM ascorbate. (B) Cells fixed and stained with streptavidin (green), DAPI (blue), and ICAM-1 (grey). Images were acquired by confocal microscopy. Scale bar, 20 μm. (C) Biotinylated proteins were pulled down using neutravidin beads, and pulldown samples were analysed by mass spectrometry. Heat map of 2 independent mass spectrometry data sets is shown with white meaning no signal and dark red a high signal. Each individual experiment was carried out in duplicate, with mass spectrometry runs being repeated twice (to give a total of 4 analyses/experiment). p-Values are given across 2 experiments (*p ≤ 0.05, **p ≤ 0.01, ***p ≤ 0.001, ****p ≤ 0.0001; t test). Cotrafficked proteins appear in both ± ascorbate conditions, whilst proteins adjacent to JAM-C solely at the cell surface are only present in the −ascorbate condition. (D and E) HUVECs were stimulated for 4 h with TNF-α fixed and labelled for (D) JAM-C (green) and VE-Cadherin (magenta) or (E) JAM-C (green) and PECAM-1 (magenta). JAM-C does not colocalise with VE-Cadherin or PECAM-1. Scale bar, 20 μm. HRP, horseradish peroxidase; HUVEC, human umbilical vein endothelial cell; ICAM, intercellular adhesion molecule; JAM-C, junctional adhesion molecule-C; PECAM-1, platelet endothelial cell adhesion molecule 1; TNF, tumour necrosis factor; VE-Cadherin, vascular endothelial cadherin. (TIF) [file pbio.3000554.s003.tif]
