# Supplementary material for: Dynamic trafficking and turnover of JAM-C is essential for endothelial cell migration
Source: PLoS Biol. 2019 Dec 2;17(12):e3000554. doi: 10.1371/journal.pbio.3000554 (PMC6907879; doi:10.1371/journal.pbio.3000554)
Supplement: S3 Table — (DOCX) [file pbio.3000554.s010.docx]

Primers for Mutagenesis

| Mutant | Primer | Sequence (5’-3’) |
| --- | --- | --- |
| Y267A | F  R | GGCATCTGCTGTGCGGCCAGACGAGGCTGCTTC  GAAGCAGCCTCGTCTGGCCGCACAGCAGATGCC |
| S281A | F  R | CAGTAAACAAGATGGAGAAGCCTATAAGAGCCCAGGGAAGC  GCTTCCCTGGGCTCTTATAGGCTTCTCCATCTTGTTTACTG |
| Y282A | F  R | GTAAACAAGATGGAGAAAGCGCTAAGAGCCCAGGGAAGCATGAC  GTCATGCTTCCCTGGGCTCTTAGCGCTTTCTCCATCTTGTTTAC |
| K283R | F  R | GATGGAGAAAGCTATAGGAGCCCAGGGAAGCATG  CATGCTTCCCTGGGCTCCTATAGCTTTCTCCATC |
| K287R | F  R | CTATAAGAGCCCAGGGAGGCATGACGGTGTTAAC  GTTAACACCGTCATGCCTCCCTGGGCTCTTATAG |
| Y293A | F  R | GCATGACGGTGTTAACGCCATCCGGACGAGTGAGG  CCTCACTCGTCCGGATGGCGTTAACACCGTCATGC |
| T296A | F  R | GGTGTTAACTACATCCGGGCGAGTGAGGAGGGTGACTTCAG  CTGAAGTCACCCTCCTCACTCGCCCGGATGTAGTTAACACC |

Infusion replacement of HRP for GFP in JAM-C GFP Out

| Protein | Primer | Sequence (5’-3’) |
| --- | --- | --- |
| HRP | F  R | GGAAGTCTATTCTAGAATGCAGTTAACCCCTACATTCTAC  CCAGCAATGTTCAACTCGAGAGAGTTGCTGTTGACCACTCTG |

Infusion subcloning of JAM-C GFP Out into lentiviral vectors

| Mutant | Primer | Sequence (5’-3’) |
| --- | --- | --- |
| WT JAM-C GFP Out | F  R | GGATCCCGGGCTCGAGATGGCGCTGAGCCGGCG  TACCAGGCCTCTCGAGTCAGATAACAAAGGACGATTTGTGT |
| Quad K JAM-C GFP Out | F  R | GGATCCCGGGCTCGAGATGGCGCTGAGCCGGCG  TACCAGGCCTCTCGAGTCAGATAACAAAGGACGATCTG |

**S3 Table Primer Sequences**
