# Supplementary material for: Dynamic trafficking and turnover of JAM-C is essential for endothelial cell migration
Source: PLoS Biol. 2019 Dec 2;17(12):e3000554. doi: 10.1371/journal.pbio.3000554 (PMC6907879; doi:10.1371/journal.pbio.3000554)
Supplement: S4 Table — (DOCX) [file pbio.3000554.s011.docx]

| **Antibody** | **Source** | **Cat #/Ref** | **Species** | **Details** |
| --- | --- | --- | --- | --- |
| HA | Roche | 11867423001 | Rat monoclonal | Western 1:250 |
| GFP | Chromotek | 3H9 | Rat monoclonal | Western 1:1000 |
| JAM-C | A kind gift from Prof Beat Imhof (University of Geneva) | J81 | Western 1:1000  IF 1:1000 | Western 1:1000  IF 1:1000 |
| JAM-C | Bethyl Laboratories | A303-761A | Rabbit polyclonal | Western 1:1000 |
| VE-cadherin | Santa Cruz | Sc9989 | Mouse monoclonal | Western 1:1000  IF 1:100 |
| ZO-1 | Cell signalling technology | D6L1E | Rabbit monoclonal | Western 1:1000  IF 1:100 |
| CD63 | AbCAM | CLB180 | Mouse monoclonal | IF 1:100 |
| Rab11 | Lifetechnologies | 71-5300 | Rabbit polyclonal | IF 1:100 |
| NRP-1 | AbCAM | Ab81321 | Rabbit polyclonal | Western 1:1000  IF 1:100 |
| NRP-2 | R &D Signalling | AF2215 | Rabbit polyclonal | Western 1:200  IF 1:50 |
| PECAM (CD31) | E-bioscience | 17-0319-41 | Mouse monoclonal (WM-59) | IF 1:200 |
| PECAM (CD31) | Santa Cruz | Sc1506 | Goat polyclonal | Western 1:1000 |
| JAM-A | Santa Cruz | Sc53628 | Mouse monoclonal | Western 1:1000  IF 1:100 |
| CD99 | Santa Cruz | Sc28389 | Mouse monoclonal (12E7) | Western 1:100 |
| Tubulin | Sigma | T4026 | Mouse monoclonal (Tub2.1) | Western 1:1000 |
| Streptavidin HRP | DAKO | P0397 | - | Western 1:1000 |
| ICAM-1 | Santa Cruz | Sc7891 | Rabbit polyclonal | Western 1:1000 |
| CBL | Santa Cruz | Sc398282 | Mouse monoclonal | Western 1:1000 |

**S4 Table Antibodies**
