# Supplementary material for: Dynamic trafficking and turnover of JAM-C is essential for endothelial cell migration
Source: PLoS Biol. 2019 Dec 2;17(12):e3000554. doi: 10.1371/journal.pbio.3000554 (PMC6907879; doi:10.1371/journal.pbio.3000554)
Supplement: S1 Raw Images — (PDF) [file pbio.3000554.s020.pdf]

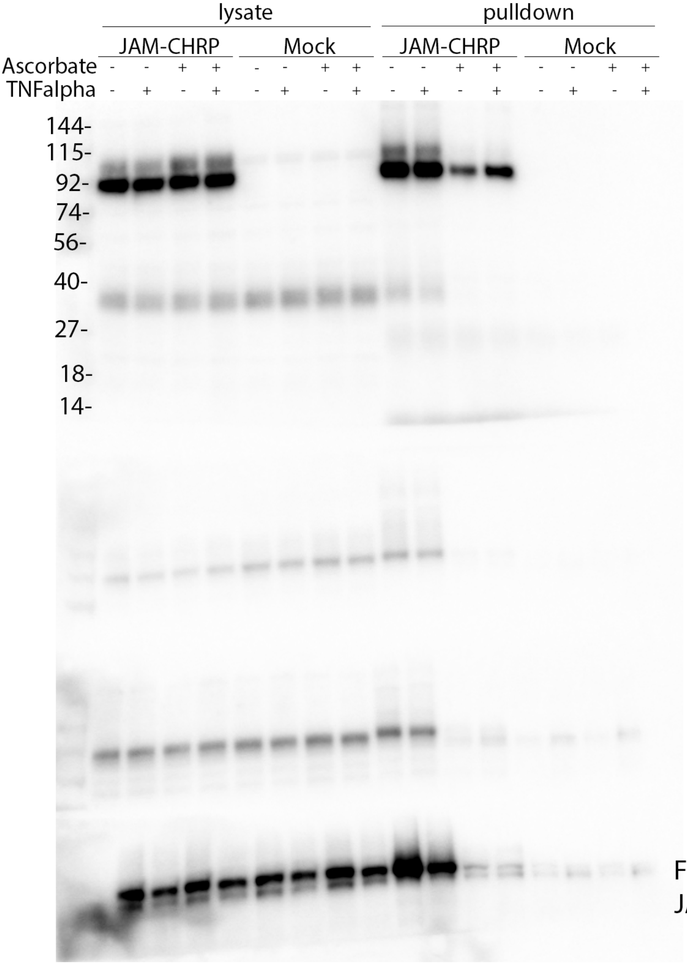
lysate
pulldown
JAM-CHRP
Mock
JAM-CHRP
Mock
Ascorbate - - + + - - + + - - + + - - + +
TNFalpha - + - + - + - + - + - + - + - +
144-
115-
92-
74-
56-
40-
27-
18-
14-
Fig.3E
JAM-C

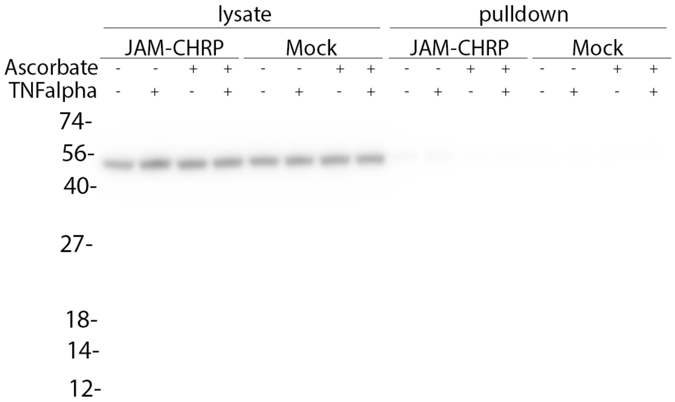

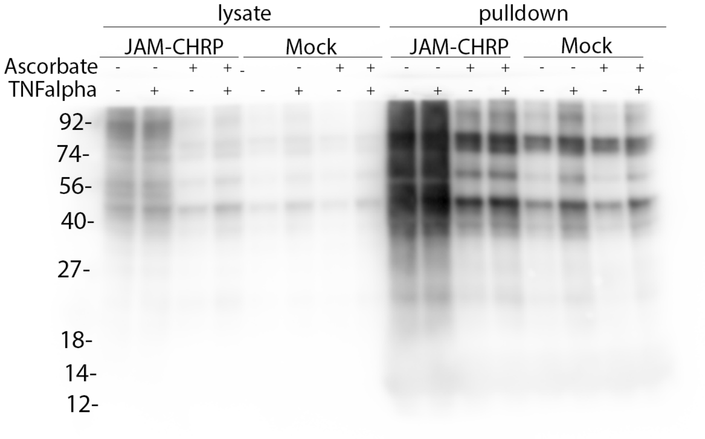

Fig.3E exp2
Tubulin-top
Streptavidin-bottom

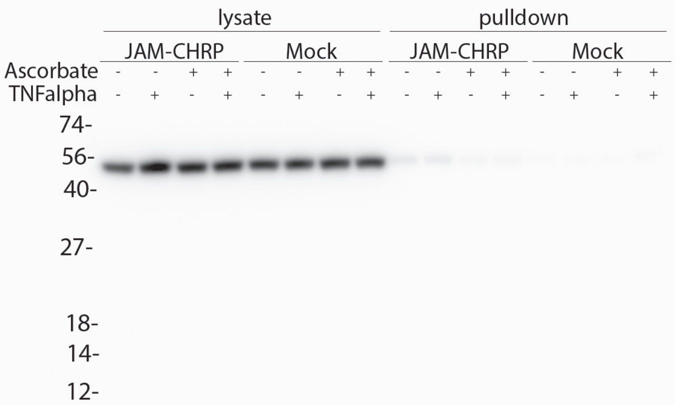

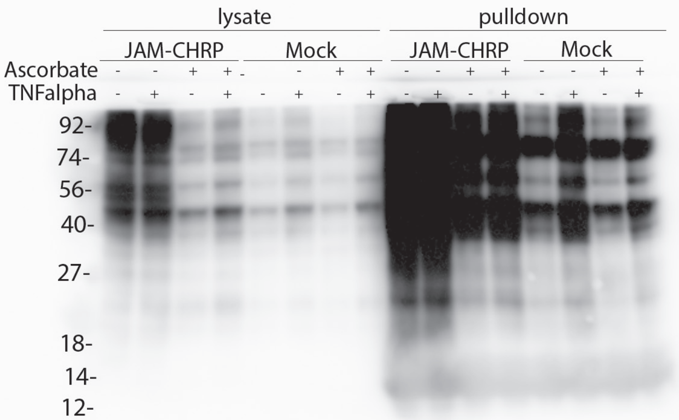

Fig.3E exp1
Tubulin-top
Streptavidin-bottom

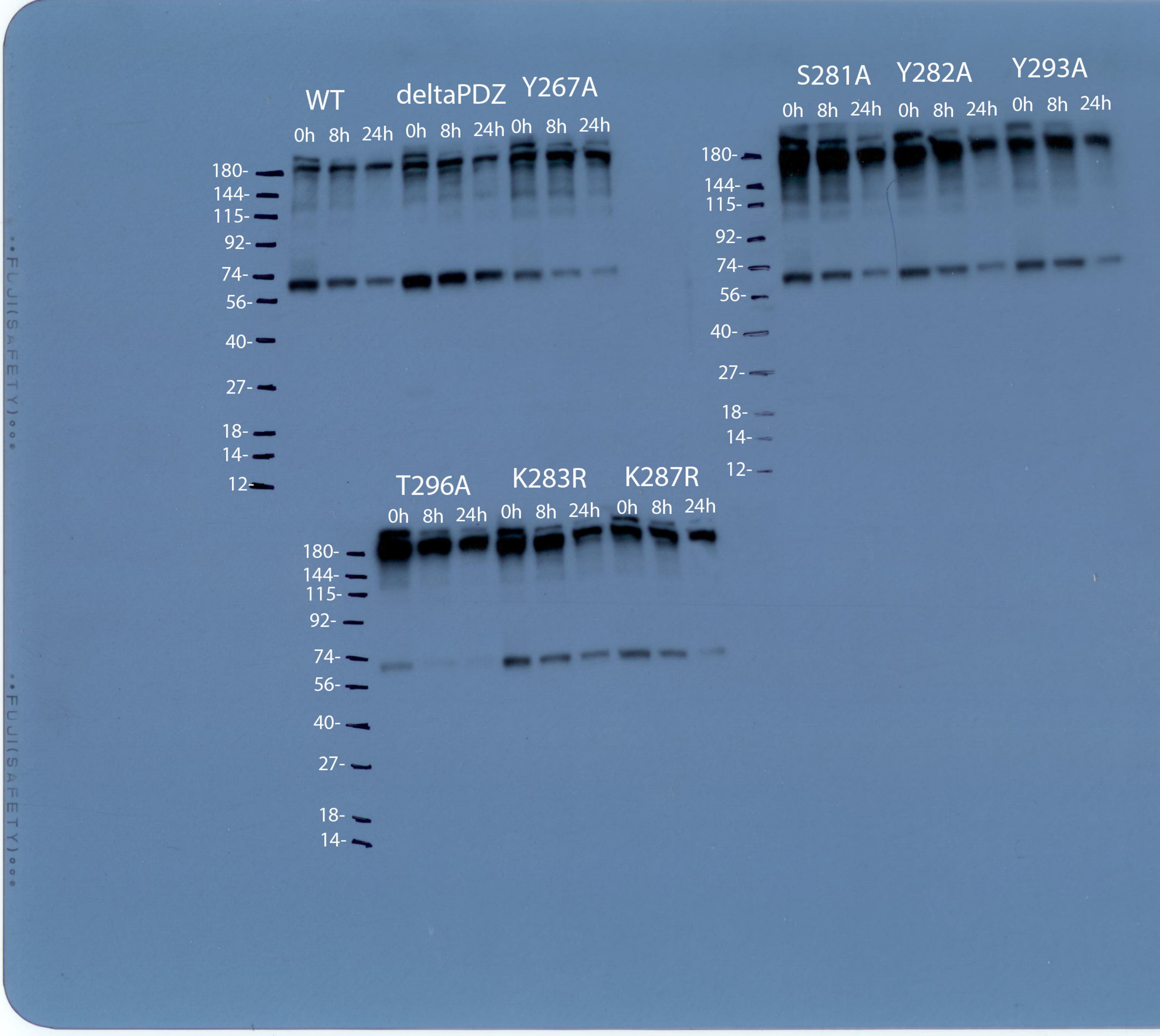
WT
deltaPDZ
Y267A
0h 8h 24h 0h 8h 24h 0h 8h 24h
180-
144-
115-
92-
74-
56-
40-
27-
18-
14-
12-
S281A
Y282A
Y293A
0h 8h 24h 0h 8h 24h 0h 8h 24h
180-
144-
115-
92-
74-
56-
40-
27-
18-
14-
12-
T296A
K283R
K287R
0h 8h 24h 0h 8h 24h 0h 8h 24h
180-
144-
115-
92-
74-
56-
40-
27-
18-
14-

Fig. 4C exp1

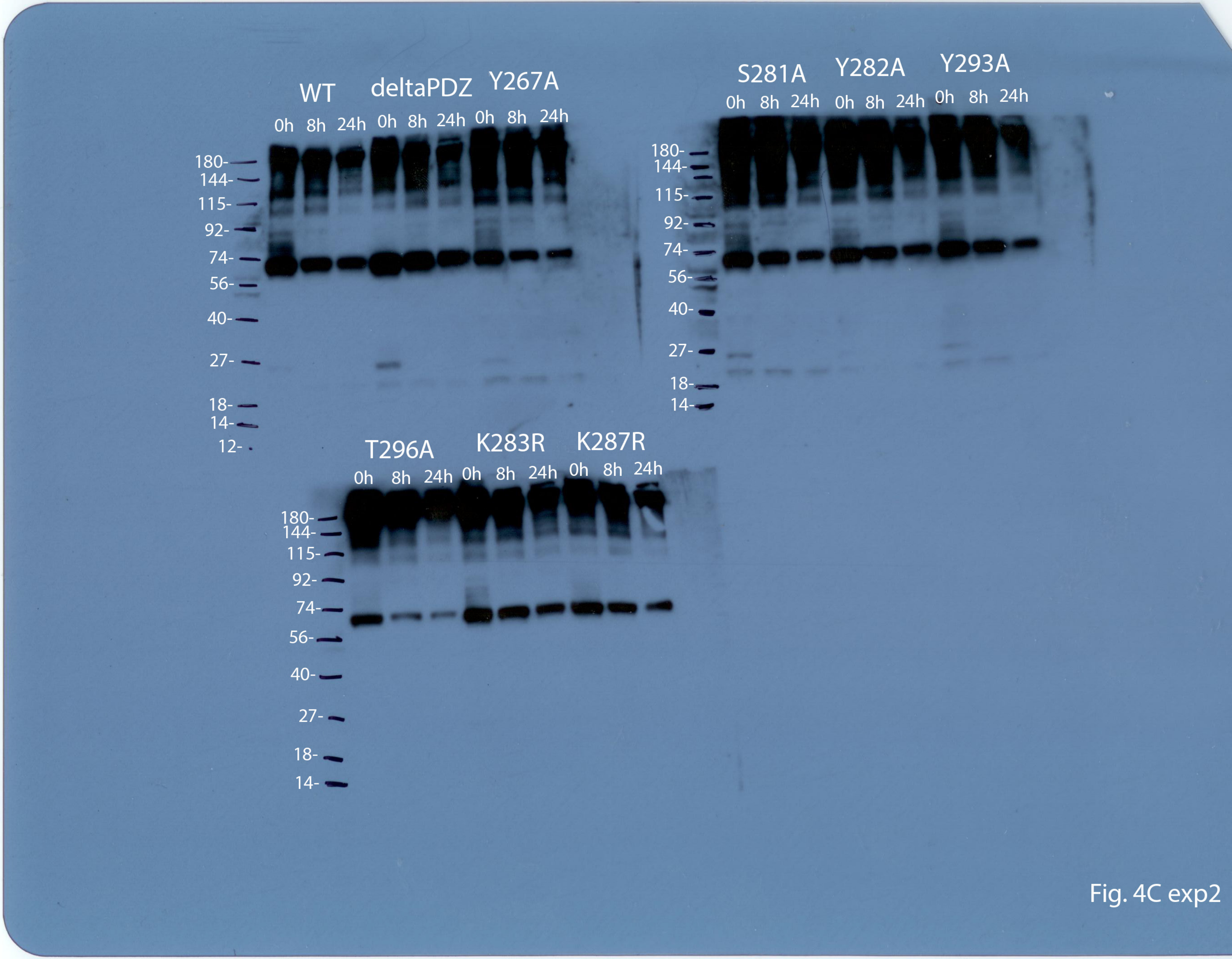
WT
deltaPDZ
Y267A
0h 8h 24h 0h 8h 24h 0h 8h 24h
180-
144-
115-
92-
74-
56-
40-
27-
18-
14-
12-
S281A
Y282A
Y293A
0h 8h 24h 0h 8h 24h 0h 8h 24h
180-
144-
115-
92-
74-
56-
40-
27-
18-
14-
T296A
K283R
K287R
0h 8h 24h 0h 8h 24h 0h 8h 24h
180-
144-
115-
92-
74-
56-
40-
27-
18-
14-

Fig. 4C exp2

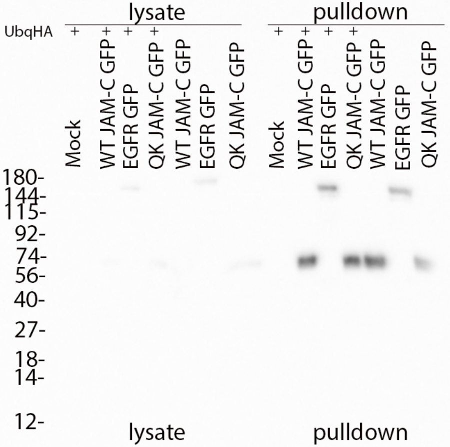

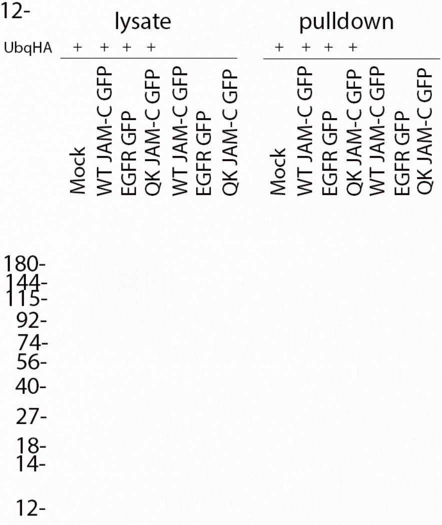

Fig.5B 1secexp

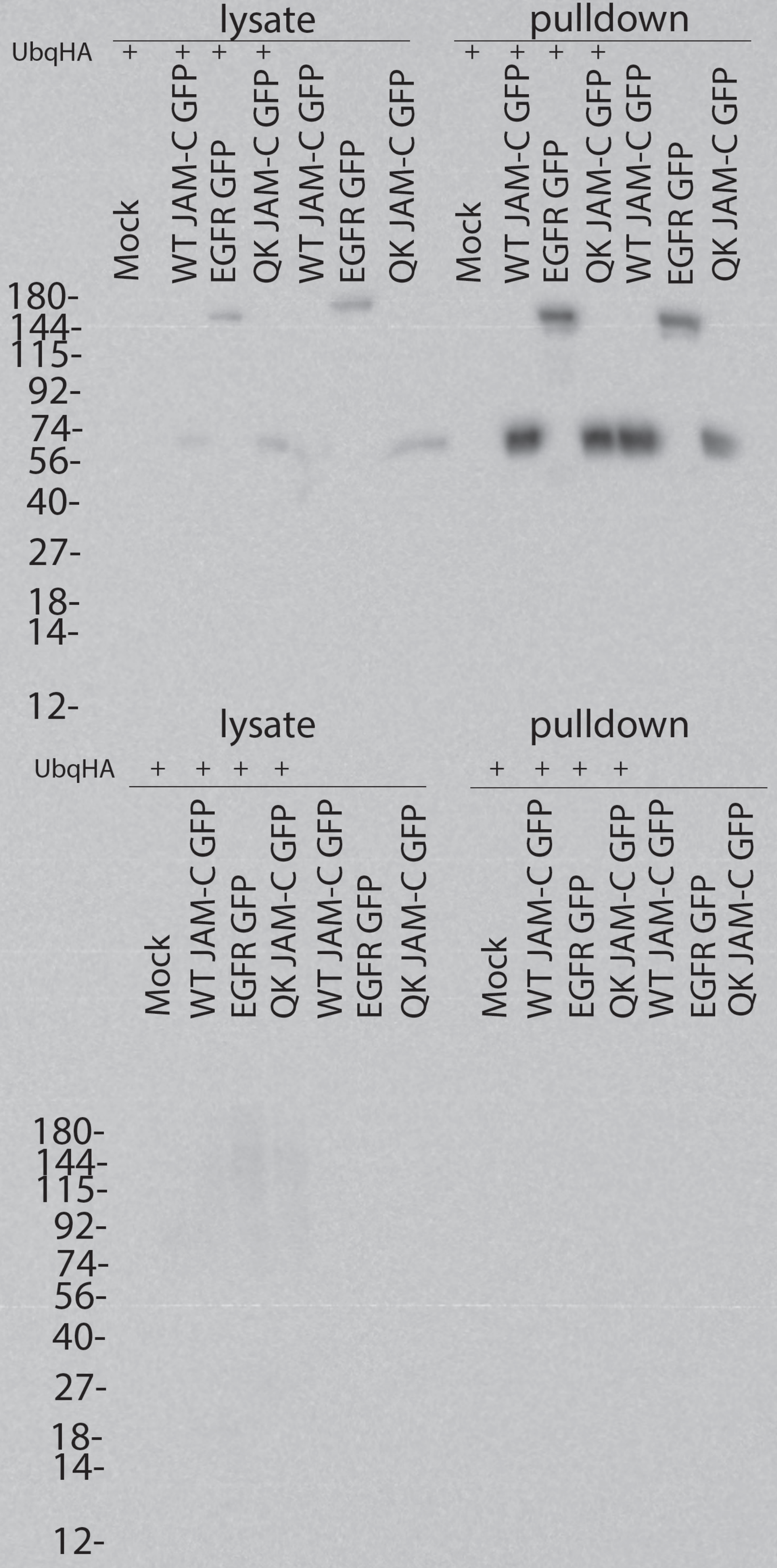

Fig.5B 1sec (background increased)

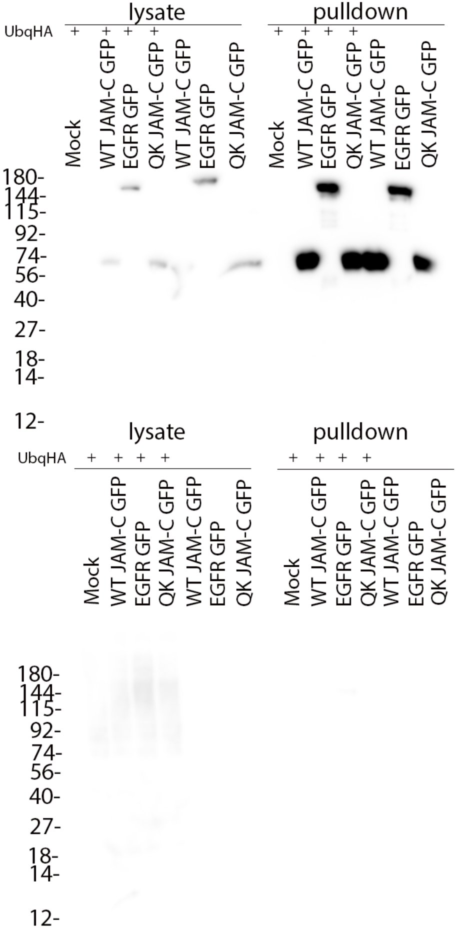
lysate
pulldown
UbqHA
Mock
WT JAM-C GFP
EGFR GFP
QK JAM-C GFP
WT JAM-C GFP
EGFR GFP
QK JAM-C GFP
Mock
WT JAM-C GFP
EGFR GFP
QK JAM-C GFP
WT JAM-C GFP
EGFR GFP
QK JAM-C GFP
180-
144-
115-
92-
74-
56-
40-
27-
18-
14-
12-
lysate
pulldown
UbqHA
Mock
WT JAM-C GFP
EGFR GFP
QK JAM-C GFP
WT JAM-C GFP
EGFR GFP
QK JAM-C GFP
Mock
WT JAM-C GFP
EGFR GFP
QK JAM-C GFP
WT JAM-C GFP
EGFR GFP
QK JAM-C GFP
180-
144-
115-
92-
74-
56-
40-
27-
18-
14-
12-

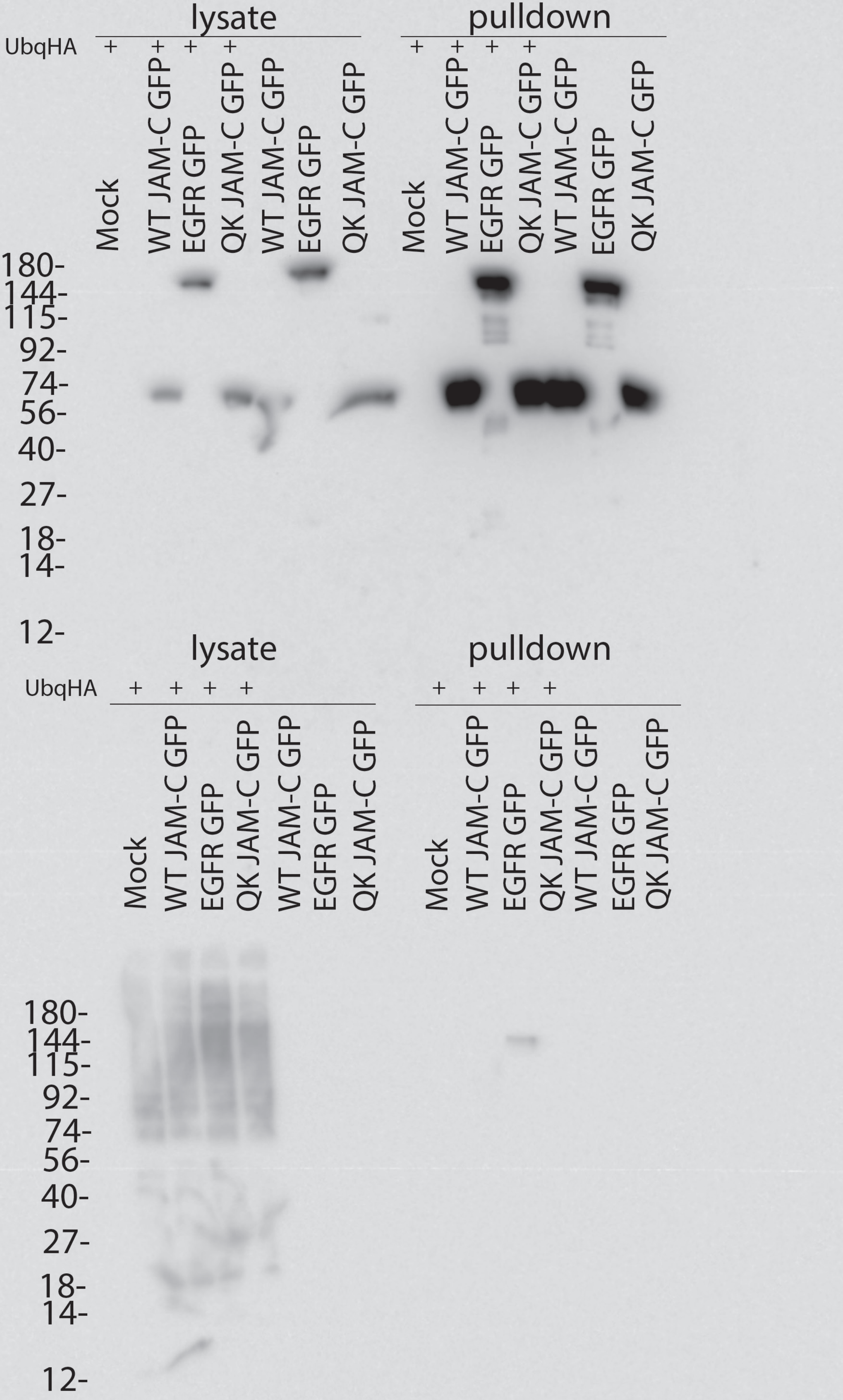

Fig.5B 1min (background increased)

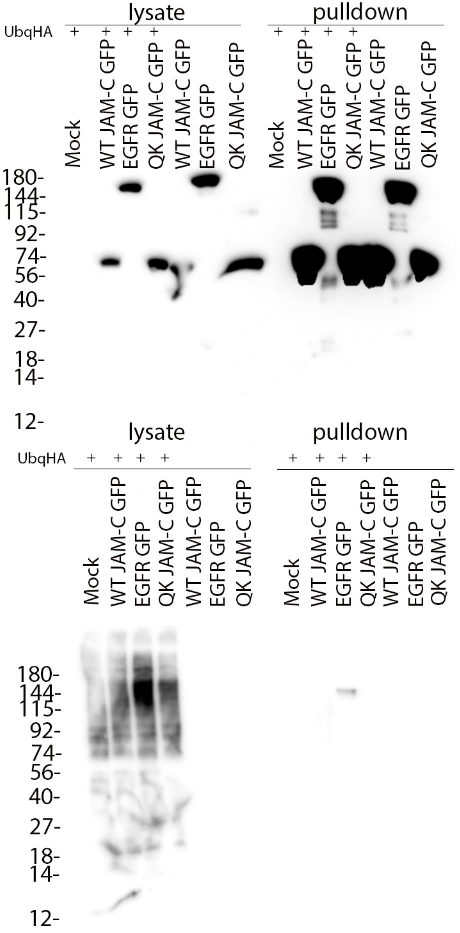

Fig.5B 18min-exp

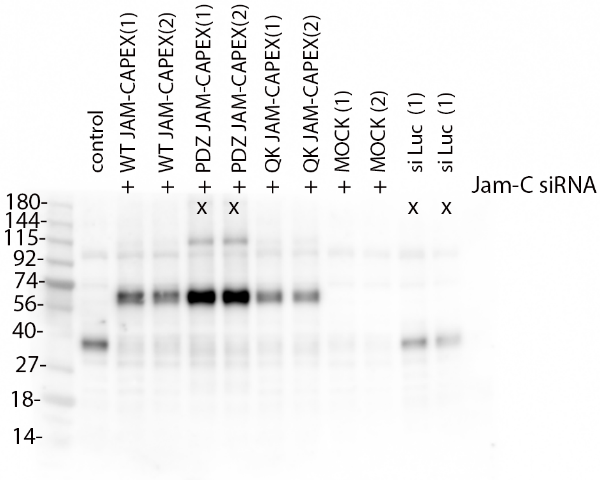
control
WT JAM-CAPEX(1)
WT JAM-CAPEX(2)
PDZ JAM-CAPEX(1)
PDZ JAM-CAPEX(2)
QK JAM-CAPEX(1)
QK JAM-CAPEX(2)
MOCK (1)
MOCK (2)
si Luc (1)
si Luc (1)
+ + + + + + + +
Jam-C siRNA
x x x x
180-
144-
115-
92-
74-
56-
40-
27-
18-
14-

Fig.6B Jam-C

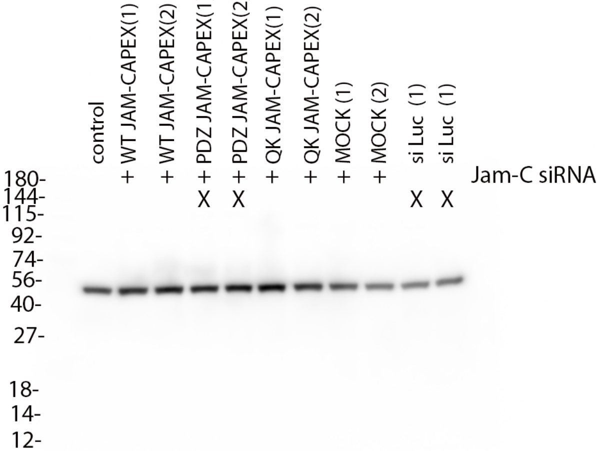

Fig.6B Tubulin

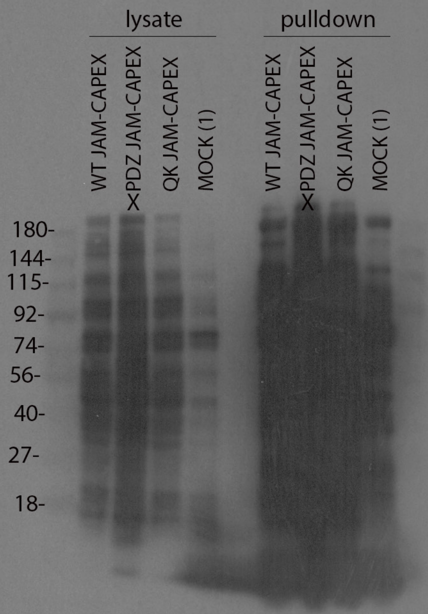

Fig.6C
Streptavidin
exp1

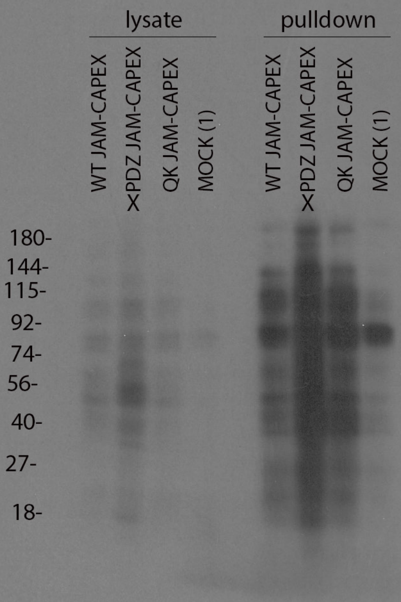

Fig.6C
Streptavidin
exp2

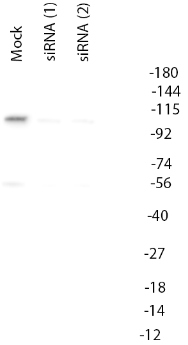

Fig. 7A Cbl blot

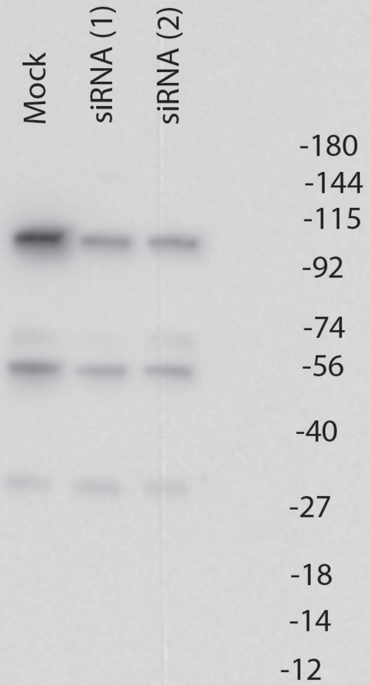

Fig. 7A Cbl blot

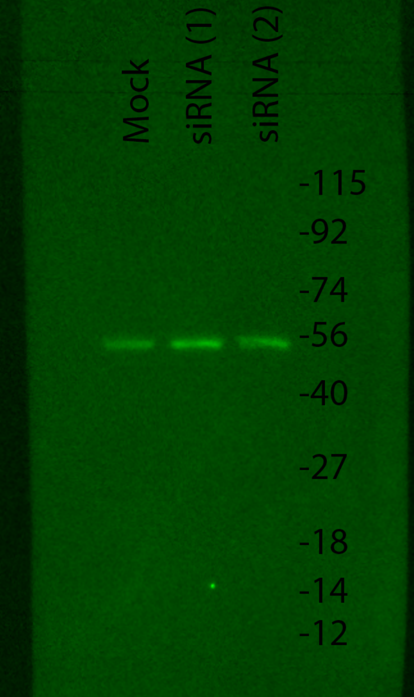

Fig. 7A Tubulinblot

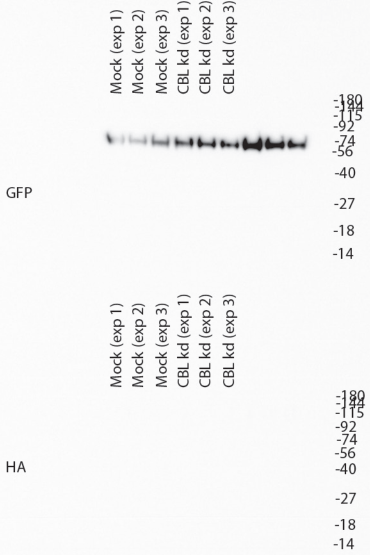
Mock (exp 1)
Mock (exp 2)
Mock (exp 3)
CBL kd (exp 1)
CBL kd (exp 2)
CBL kd (exp 3)
-180
-144
-115
-92
-74
-56
-40
GFP
-27
-18
-14
Mock (exp 1)
Mock (exp 2)
Mock (exp 3)
CBL kd (exp 1)
CBL kd (exp 2)
CBL kd (exp 3)
-180
-144
-115
-92
-74
-56
HA
-40
-27
-18
-14

Fig. 7B exp 1

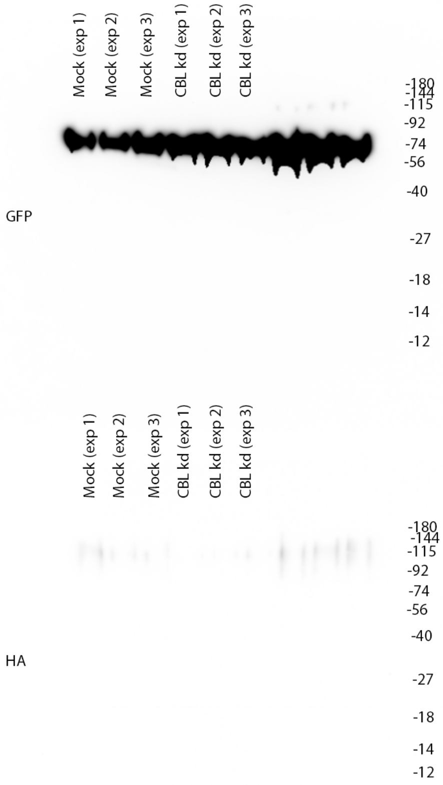

Fig. 7B 55 min exp

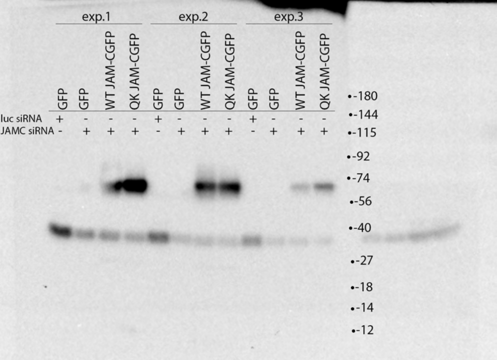

Fig. 8A JAM-C blot

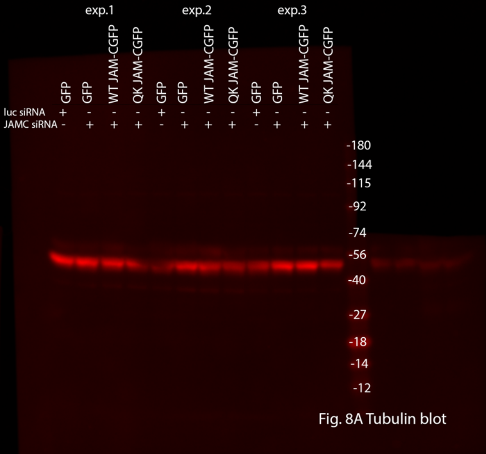

Fig. 8A Tubulin blot

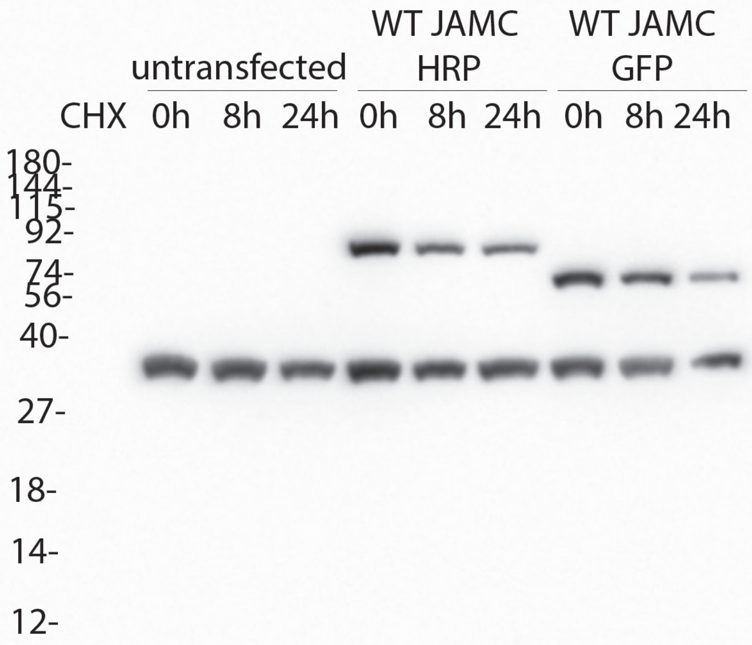

Supp. Fig. 1A JAM-C blot

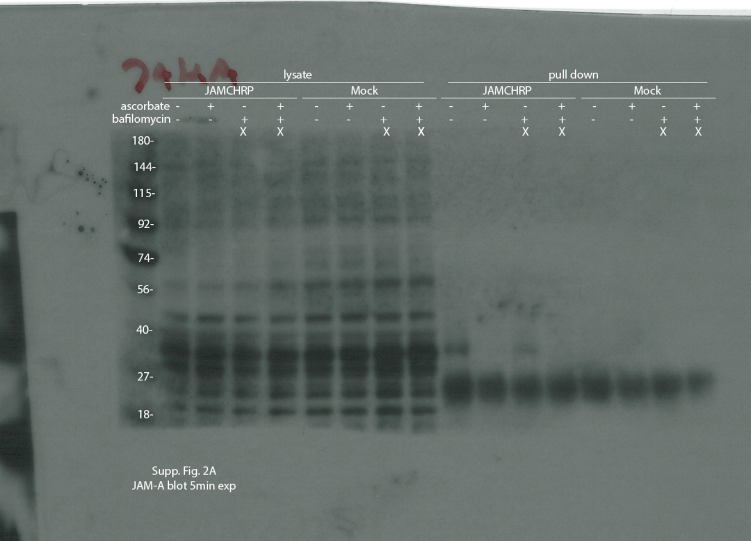

Supp. Fig. 2A
JAM-A blot 5min exp

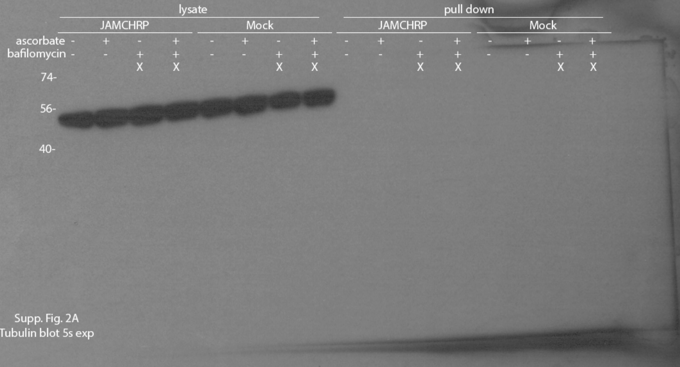

Supp. Fig. 2A
Tubulin blot 5s exp

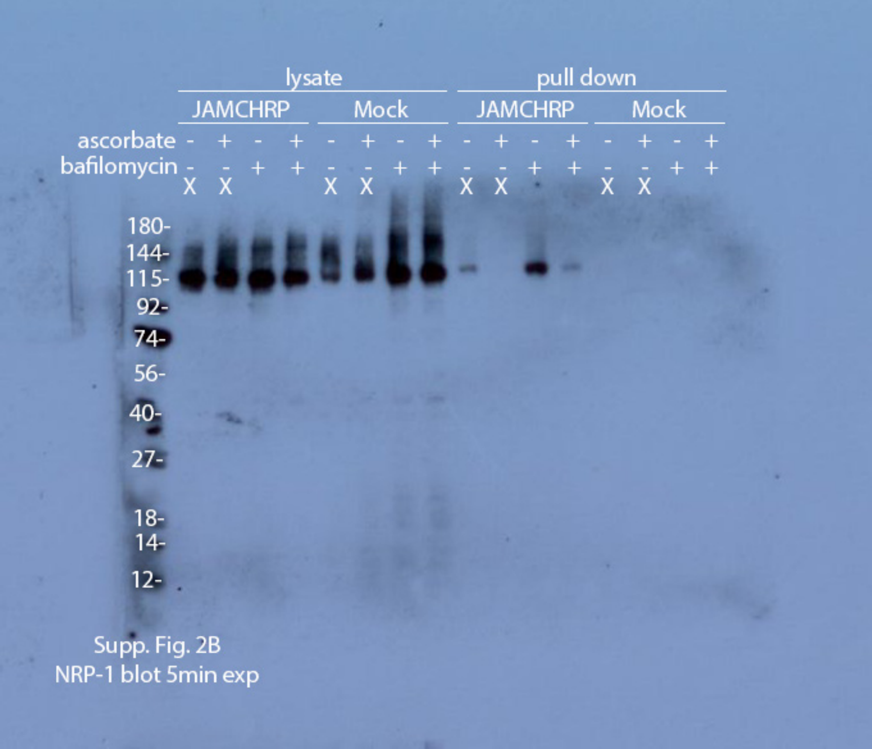
lysate
pull down
JAMCHRP
Mock
JAMCHRP
Mock
ascorbate - + - + - + - + - + - + - + - +
bafilomycin - - + + - - + + - - + + - - + +
X X X X X X X X
180-
144-
115-
92-
74-
56-
40-
27-
18-
14-
12-
Supp. Fig. 2B
NRP-1 blot 5min exp

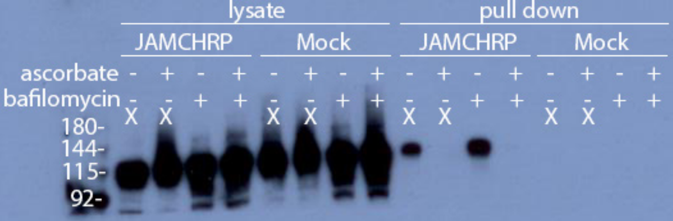

Supp. Fig. 2B
NRP-2 blot 5min exp

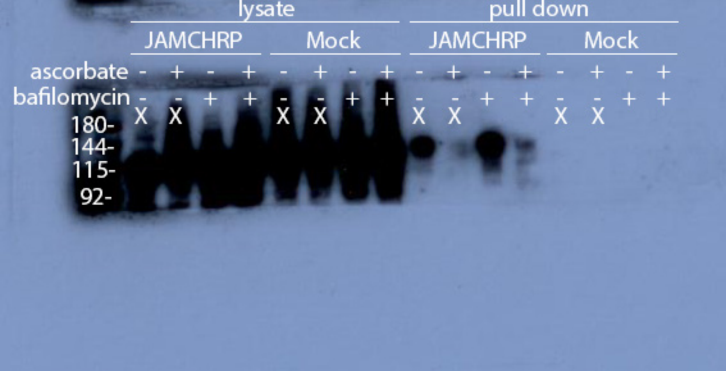

Supp. Fig. 2B
NRP-2 blot 30min exp

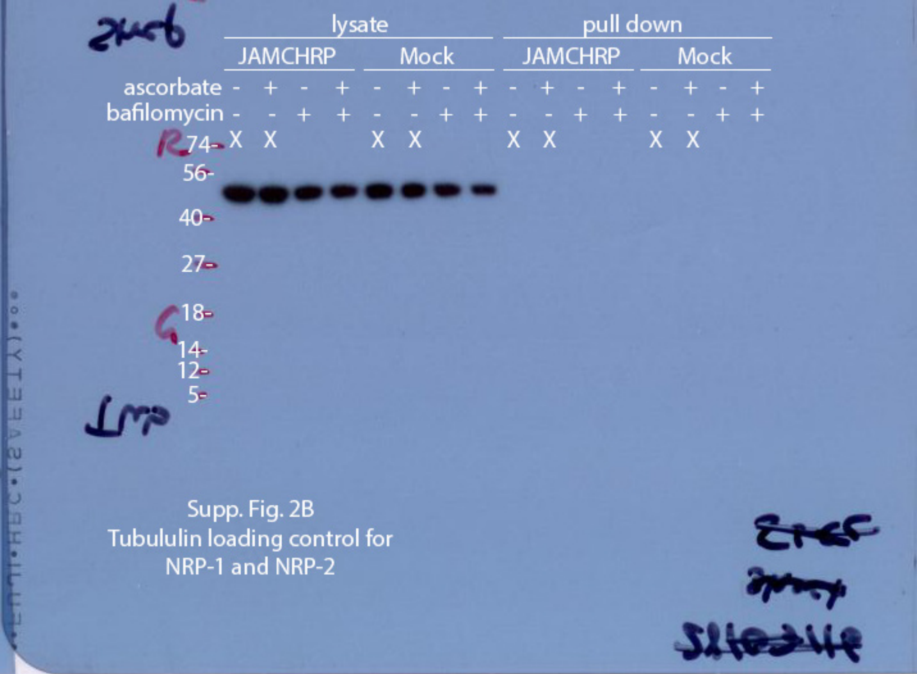

Supp. Fig. 2B
Tubululin loading control for
NRP-1 and NRP-2

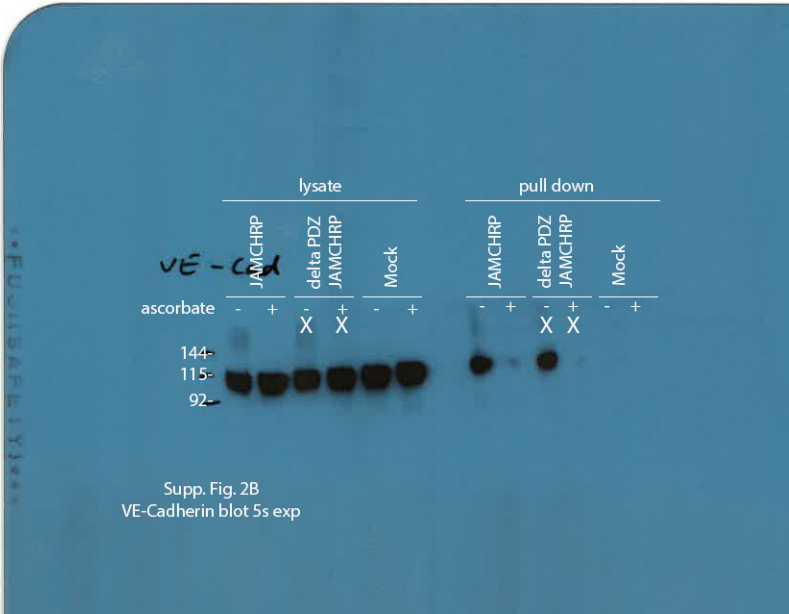

Supp. Fig. 2B
VE-Cadherin blot 5s exp

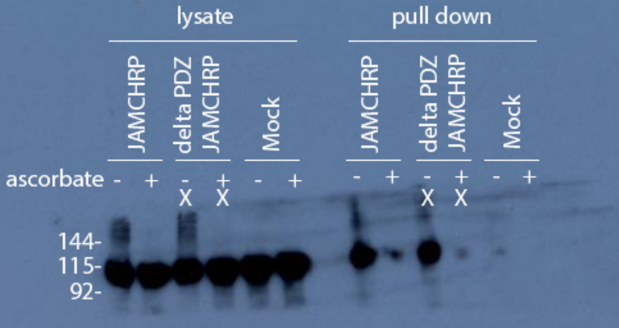

Supp. Fig. 2B
VE-Cadherin blot 30s exp

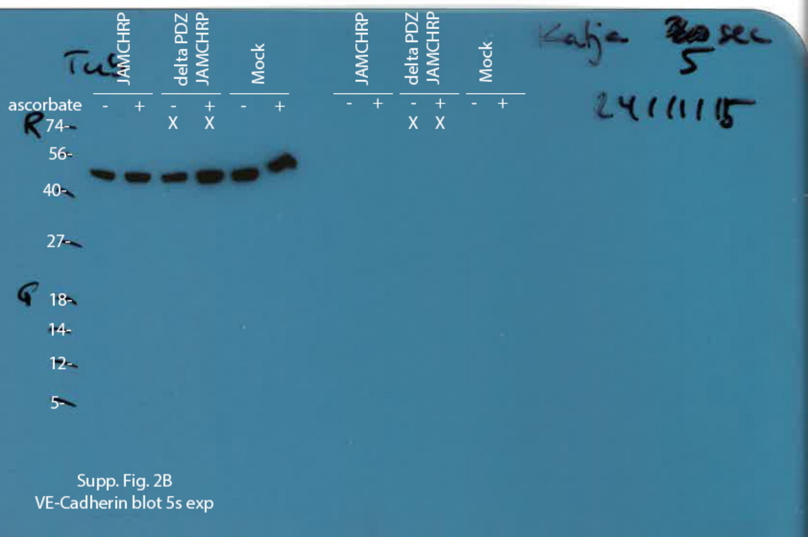

Supp. Fig. 2B
VE-Cadherin blot 5s exp

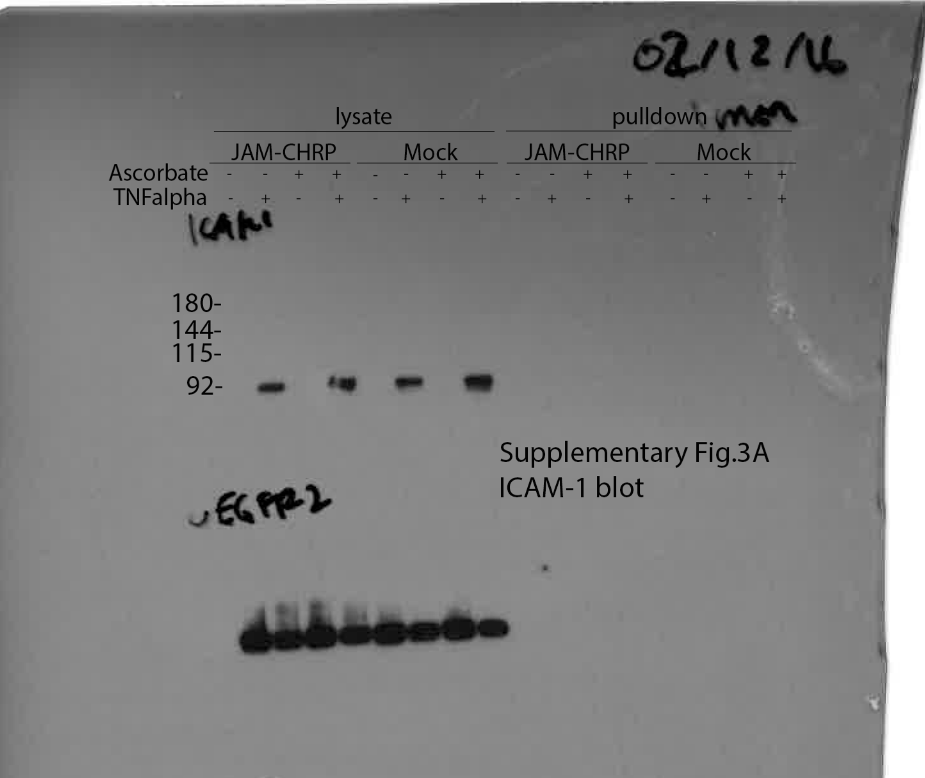

Supplementary Fig.3A
ICAM-1 blot

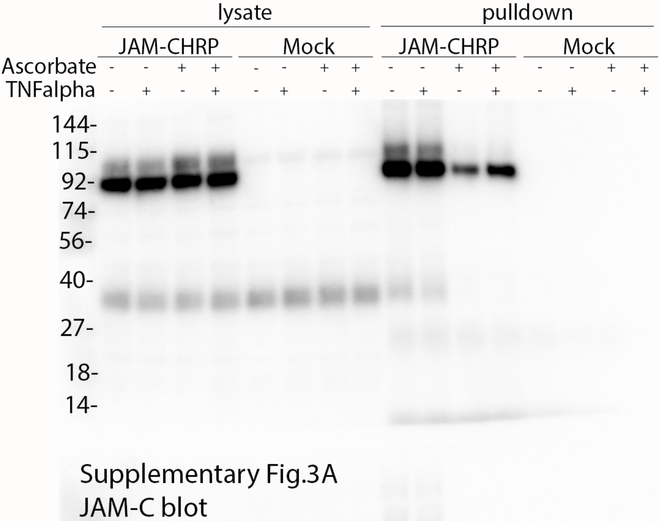

Supplementary Fig.3A
JAM-C blot

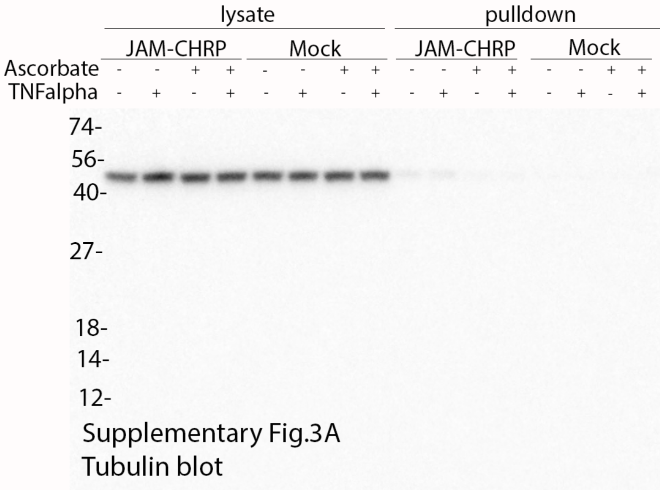

Supplementary Fig.3A
Tubulin blot
