# Supplementary material for: Dynamic trafficking and turnover of JAM-C is essential for endothelial cell migration
Source: PLoS Biol. 2019 Dec 2;17(12):e3000554. doi: 10.1371/journal.pbio.3000554 (PMC6907879; doi:10.1371/journal.pbio.3000554)
Supplement: Attachment — Submitted filename: Response to Reviewers.docx [file pbio.3000554.s021.docx]

Response to Reviewers

We thank the reviewers for taking the time to read the paper and for their comments. We have addressed their concerns with new experiments and amendments to the paper, all of which we feel has significantly improved the publication.

Specific points below:

Reviewer 1.

*The reviewer states “The major issue is that the authors assume that the pathway taken by JAM-C-HRPout is the same as taken for native JAM-C…However, they never studied turnover of the construct.  JAM-C has an apparent Mr of 29 - 45 kD depending on the report.  HRP has an Mr of 44 kD.  It is quite possible that placing a molecule the size of JAM-C or larger into the JAM-C extracellular domain would affect its turnover and its ability to recycle.”*

We do agree with the reviewer that tagging JAM-C, like tagging any protein, may change the attributes of that protein. We have included additional analysis using a cycloheximide chase to monitor how the turnover of JAM-C varies following tagging in the membrane proximal domain of JAM-C with GFP, HRP and compare this to the endogenous JAM-C (see Supp. Fig. 1A & B). There is no significant difference in either of the tagged constructs compared to the endogenous JAM-C. The strongest evidence that tagged JAM-C traffics appropriately is from the functional data. We can rescue migration defects to almost endogenous levels with WT tagged JAM-C, but not with the Quad K mutant tagged JAM-C (Fig.8) that does not exhibit normal trafficking (Fig. 4 and 5). We have amended the text to acknowledge the reviewer’s concerns (lines 129 to 134) as they are important caveats to bear in mind when interpreting our presented data.

*Fig. 2 The antibody against JAM-C used in A-C is a polyclonal antibody, which can cross-link JAM-C and increase its rate of internalization.  What happens if a monoclonal antibody is used?*

Antibody feeding is utilised for Fig. 2A and B to show endogenous cell surface JAM-C can rapidly traffic intracellularly. Fig. 2C and D are images generated by immunofluorescence labelling and confocal microscopy, the legends have been amended to make this clearer. Antibody mediated cross linking of cell surface receptors can affect rate of internalisation and activate receptors [1] and this is particularly true of polyclonal antibodies [2]. We unfortunately don’t have a suitable monoclonal antibody to human JAM-C to compare to the polyclonal antibody used in this study. We have tested almost all of the commercially available JAM-C antibodies both mono and polyclonal and none are suitable for immunofluorescent labelling. In the manuscript the antibody feeding experiment is corroborated by live cell imaging of tagged receptor (Fig. 2G) and immunofluorescence analysis of the accrual of lysosomal targeted receptor (following bafilomycin treatment). From the published literature and by comparison to CHX chase and immunofluorescence analysis it is likely the rate of JAM-C trafficking is higher following antibody feeding with the polyclonal antibody. We have amended the text to acknowledge this (lines 144 to 147) the experiment does however demonstrate that endogenous JAM-C traffics internally.

*Fig. 3E.  What is “mock” conditions?  I can’t find this explained anywhere.  Why is so much material pulled down under “mock” conditions in E but not seen in the corresponding proteomic experiments in 3F?*

Apologies this should have been clearer. Mock conditions represent HUVEC nucleofected with nucleofection buffer and then treated with fed biotin phenol in the presence of hydrogen peroxide. We have amended the methods to better detail this (lines 607-608 and 650-656).

The bands shown in the mock indicate that some endogenously biotinylated proteins and/or background biotinylation are present even in mock conditions [3]. No background is shown in the mass spectrometry data presented in Fig. 3F as this focuses on the receptors that are co-trafficked; are significantly different from the mock and at least 3.5 fold enriched. A lower resolution map of the data from one round of mass spectrometry has been added to the supplementary data Supplementary Fig. 1D to show the spread of the mass spectrometry data and the background mass spectrometry results present. The results have been improved to make this distinction clearer (lines 257 to 260).

*Figs. 5, 6, 7  Considering that ubiquitylation of membrane proteins is a well-known and accepted mechanism for targeting them for degradation, the data of these figures does not seem very surprising or significant compared to the proteomic data of Fig 3.  The E3 ligase for JAM-C was not previously known, but it is not surprising that one exists.  The authors could consider shortening this part of the manuscript or relegating it to Supplementary Materials and focus on the significance of their primary observation and the experiment of Figure 8.*

We understand the reviewer’s opinion with regards to Figs. 5-7 but disagree with the description of its significance. In our opinion the paper shows the importance of JAM-C receptor trafficking to its function in cell migration. To do this it is essential to define at least some of the regulatory mechanisms of trafficking. Only then can the process be selectively perturbed and its importance demonstrated as in Fig. 8. The role of ubiquitylation in trafficking, the means of identifying trafficking machinery, and the E-3 ligase CBL are all novel. CBL, for example, has never been shown to ubiquitylate a junctional receptor before.

*Fig. 8 shows that inhibition of ubiquitination and turnover of JAM-C blocks migration.  The authors propose that this is due to more JAM-C at the junctions.  Previous figures show that blocking ubiquitylation of JAM-C increases JAM-C-GFPout levels and decreases the number of fluorescent vesicles, but the intensity of JAM-C at the junctions does not appear to be different.  Can the authors quantify junctional JAM-C?*

We have quantified the amount of JAM-C at endothelial cell junctions in the CBL knock down cells and cannot show a significant difference in intensities. This may be because there is an incomplete inhibition of the E-3 ligase (CBL) or because there is redundancy with another E-3 ligase. Segmentation of internal vs cell surface receptor is more complicated in immunofluorescent labelled samples compared to the antibody feeding assay and a better biochemical way to characterise surface pools is required. As the quantification of junctional levels is inconclusive we have not included it in the manuscript but we have presented it below for the reviewers to assess. We have included further discussion about the cell surface levels (lines 419 to 421 in the results and lines 562 to 566 in the discussion).

*Fig. 9 The video provided does not make a good case that internalization of JAM-C-GFPout is at the sides of the cells as opposed to at the front of the migrating cell.*

We have included new movies to show the manner in which Jam-C traffics as cells migrate to fill a scratch wound. We have also softened our conclusion see changes in the results and discussion (results lines 478 to 480 and discussion lines 562-566).

*Almost none of the western blots have molecular size labels. They should.*

We have added molecular weights to all the blots.

*Fig. 1 Were the cells permeabilized to be able to see punctae?*

The cells were permeabilised we have amended the methods to make this clearer (line 716).

*Line 297  TNFa is a known permeability agent; IL-1b is not.  Therefore, instead of being stimulus specific, a more circumspect interpretation would be that this change is seen with treatments that reduce barrier function.*

We agree with the reviewers comment and have amended the text accordingly (lines 188 to 189).

*It is a bit disingenuous in the schematic diagrams in multiple figures to represent the HRP and GFP as small circles compared to the large JAM-C sausage, when in fact HRP and GFP are about the same size or larger than the JAM-C extracellular domains.  They should be drawn to scale.*

We have amended the figures to more accurately reflect the size of the tags and the proteins.

*I am curious why the manuscript refers to the molecule as “JAM-C” when the rest of the literature as well as Dr. Aurrand-Lions, one of the pioneers of this molecule, calls it “JAM-C”.*

We have amended the nomenclature accordingly.

Reviewer 2.

*1) The HRP based proximity labeling assay was used in combination with streptavidin/biotin based pull down experiments and proteomics analysis to identify a series of proteins which are candidates for close neighbors within the vicinity of JAM-C. Verification of these candidates was attempted, but the results in supplementary figure 2 are not convincing. The proteomics results suggest that biotinylation of VE-cadherin, NRP-1 and NRP-2, triggered by JAM-C-HRP, is not completely killed by ascorbate, thus it occurs at the cell surface and in intracellular vesicles. This is indeed verified in suppl. figure 2B. However, the proteomics data suggest, that biotinylation of PECAM-1 and CD99 was not seen at all and for JAM-A there was a weak signal without ascorbate and no signal with ascorbate. The note “bands not visible due to low antibody sensitivity” as stated within the figure is not convincing. As it stands right now, no statement can be made about PECAM-1 and CD99 (they can
even not be considered as confirmed candidates for proteins in the vicinity of JAM-C) and for JAM-A the overall signal (outside plus inside the cell) is so weak that a comparison between outside and inside (with and without ascorbate) cannot be made. The results shown in suppl. figure 2A have to be done in a more sensitive way to obtain convincing and meaningful results.*

We do agree with the reviewer that the results for PECAM and CD99 by western blot are inconclusive as we could not detect a signal in the pull downs in either the + or – ascorbate conditions. This approach is very much governed by the sensitivity of the antibodies and we have removed them from the figure. We do regard the JAM-A blot as specific and importantly it shows a different banding pattern from the neuropilins and VE-Cadherin. We did also verify the lack of PECAM co-trafficking (Supp. Fig. 2D) by immunofluorescent analysis, PECAM is not present in JAM-C positive vesicles. Unfortunately our antibodies to CD99 and JAM-A are not suitable for immunofluorescence labelling. Overall, alongside the streptavidin labelling immunofluorescence and mass spectrometry we feel there is a convincing case to support our approach.

*2) For several experiments “mock” conditions were shown, but it was not stated what these negative control conditions were. For example, Fig. 3E and 3F, does mock mean a mock transfection with a JAM-C without HRP? Same for Fig. 6C and 6E. What about suppl fig. 2A and B, does “mock” mean no hydrogen peroxide, or no JAM-C-HRP or no biotinylation reagent?*

Apologies for this we have amended the methods to make this clearer. The mock conditions include biotin phenol and hydrogen peroxide but not the JAM-C HRP (Fig. 3E and F) or JAM-C APEX-2in (Fig. 6C and E) construct (See lines 607-608 and 650-656)

*3) Silencing of JAM-C did not lead to enhanced expression levels of JAM-C at cell contacts (Fig. 7F). Why then do the authors discuss that “the change in receptor recycling could prematurely increase the strength of homo and heterotypic junctional interactions with neighboring cells preventing cell movement” (bottom of page 17)? This argument should probably be omitted. Furthermore, the cartoon in figure 9B also seems to suggest that inhibition of JAM-C ubiquitylation would lead to more JAM-C at cell contacts, but this is not observed in reality (Fig. 7F). Therefore, the cartoon should be omitted or redrawn in a way, that is not in conflict with the results of figure 7F. Furthermore, the discussion should mention that lack of JAM-C ubiquitylation might affect cell migration by affecting integrins. In the light of their results showing co-trafficking of integrins with JAM-C and in the light of publications analyzing possible interactions between JAM-C and integrins (such as
reference 13 and 48), these are obvious possibilities.*

We have amended the cartoon. We agree with the reviewer that we don’t have direct evidence of more JAM-C being recycled following CBL knock down. This could reflect the partial nature of the knock down (there is likely to be residual CBL present) or there could be compensatory effects of another ligase that partially rescues the trafficking. We do have evidence that Quad K JAM-C migrates en masse into intracellular pools and that these return to the cell surface (Fig. 5E). We have removed the reference to increased levels of JAM-C at the junction. We speculate that the cell migration defects of the Quad K mutant could reflect miss-targeting or miss-timing of Jam-C transport. The results (line 419-421), the discussion lines (562-566) and the figure have been amended (Fig. 9B).

The potential role of Jam-C trafficking regulating integrin function or localisation is a very good point. We have added it to the discussion and amended the model to include this possibility (lines 572-575).

Reviewer 3.

*The authors conclude that JAM-C recycling is important, however there is no direct evidence to suggest that this is the case. Instead all experiments have been focused on internalization, ubiquitination and proteosomal degradation. Therefore statements along those lines should be avoided. Or the authors have to try to proof that JAM-C is recycled toward the plasma membrane and that this is the mechanism how JAM-C promote scratch wound migration.*

We agree with the reviewer we don’t have direct evidence of the role of recycling. The only evidence we have is indirect, from the imaging phenotype of the JAM-C Quad-K mutant. This mutant mobilises from internal pools and as we know it isn’t degraded, from the electron microscopy and the knock down experiments, we presume it is recycled. We have amended the discussion to make it much clearer (lines 562-566). In the future we aim to more directly address this point by developing a robust recycling assay.

*Figure 7: Is internalization of endogenous JAM-C affected by CBL? Pulse chase experiments with the anti-JAM-C antibody should be compared in control and siRNA treated HUVECs.*

The antibody feed experiments were utilised to demonstrate that Jam-C dynamically traffics. As we are using a polyclonal antibody it is also likely increasing the rate of traffic (see amended results lines 144-147). We don’t have an appropriate monoclonal or Fab fragment for this assay. This makes an assay for determining the rate of Jam-C internalisation following CBL knock down problematic to interpret. We hope to develop a more suitable biochemical assay to more directly asses this.

*Is the quad-K mutant of JAM-C able to rescue migration defects in CBL knockdown cells?*

We have noted a migration defect in CBL knockdown cells. However, CBL targets a number of receptors for ubiquitylation these include VEGFR2 [4]. We wouldn’t therefore necessarily expect JAM-C or the QuadK mutant to be able to rescue this phenotype.

*Please confirm that the Quad lysine mutated JAM-C variant is not ubiquitinated anymore by CBL.*

We can confirm the Quad K JAM-C mutant exhibits no ubiquitination (Fig. 6) and is not modifiable by CBL in HUVEC. We don’t have the facility to produce the individual purified components so we have not been able to verify this in a reductionist biochemical assay.

*Figure 9 and text page 15: “We noted large JAM-C positive vesicles forming at junctional sites on either side of the cell’s leading edge”.  Although the data in this figure indeed indicate that JAM-C is internalized in leader cells in the scratch assay, there is no comparison made with follower cells or cells in non-migrating endothelial cells to be able to determine whether JAM-C internalization is specific for, or enriched at the leading edge.*

We have repeated the experiment from Fig. 9A using lentiviral transduced cells this allows analysis of both cells from the leading edge and follower cells. Vesicle formation is most apparent from junctions that are remodelling and more junctional disassembly is apparent in cells near the leading edge. Vesicle transport precedes junctional disassembly in all cells.

*Does mutating the lysines in JAM-C tail affect only intracellular trafficking or also overall surface levels of JAM-C? Please analyse the amount of JAM-C surface levels of the wild type protein versus the Quad-K mutant.*

We have performed surface biotinylation experiments to compare steady state surface levels of WT and Quad K Jam-CGFP (n=3). The normalised data indicates lower levels of the Quad K receptor at the cell surface compared to WT receptor and this is significant (t-test). This lower level may reflect the pool of receptor trafficking through intracellular pools (that is unable to degrade) or it may reflect additional differences in the rate of passage through the secretory pathway. As mentioned previously recycling assays may allow better characterisation of the precise differences in WT vs QuadK JAM-C traffic.

*The authors claim to have shown a new trafficking machinery in control of JAM-C. Although the mass spectrometry results indeed have identified many proteins to be in the vicinity of JAM-C, the study continues only with knockdown studies with CBL. It is more appropriate to mention that CBL-mediated ubiquitination controls JAM-C. Unless the authors can show that other identified trafficking regulators are functionally involved in controlling trafficking of JAM-C.* 

We do agree with the reviewer we have only shown CBL is bona fide trafficking machinery and we have amended the text to make it clearer that the other proteins identified are currently only candidate machinery (see line 27 in the introduction).

*Figure 2A, B: is the antibody used to determine kinetics of JAM-C internalization not interfering with the function of JAM-C? Analysis of VE-cadherin stainings and scratch wound migration the presence of the antibody should be provided to confirm that the analysis based on this antibody closely resembles trafficking of the endogenous JAM-C protein.*

Antibody feeding is utilised for Fig. 2A and B to show endogenous cell surface JAM-C can rapidly traffic intracellularly. Antibody mediated cross linking of cell surface receptors can affect rate of internalisation and activate receptors [1] and this is particularly true of polyclonal antibodies [2]. We unfortunately don’t have a suitable monoclonal antibody to human JAM-C to compare to the polyclonal antibody used in this study. We have tested almost all of the commercially available JAM-C antibodies both mono and polyclonal and none are suitable for immunofluorescent labelling. In the manuscript the antibody feeding experiment is corroborated by live cell imaging of tagged receptor (Fig. 2G) and immunofluorescence analysis of the accrual of lysosomal targeted receptor (following bafilomycin treatment). From the published literature and by comparison to CHX chase and immunofluorescence analysis it is likely the rate of JAM-C trafficking is higher following antibody feeding with the polyclonal antibody. We have amended the text to acknowledge this (lines 144-147) the experiment does however demonstrate that JAM-C can traffic internally.

*Figure 2E: Is the observed increased number of Jam-C positive vesicles upon TNF treatment due to decreased proteosomal degradation or due to increased internalization of Jam-C from the plasma membrane?*

We cannot answer this question precisely. We see more vesicles of Jam-C following TNF alpha stimulation but we don’t see more vesicles accruing following BAF treatment and inhibition of lysosomal degradation. This is difficult to interpret and could reflect changes in recycling, internalisation or both. We have included the data for the rebuttal but do not feel this is conclusive enough to include in the paper as we are yet to develop a recycling assay.

*Figure 2G: The authors conclude that TNFa enhances internalization of Jam-C in Fig 2D. What happens to the dynamics of Jam-C-GFP after treatment with TNFa, are more membraneous structures observed under those conditions and does that explain the increased number of Jam-C vesicles?*

We do see more vesicles by live cell imaging following TNF alpha treatment. We think that this does reflect a greater rate of trafficking of vesicles but as mentioned above a recycling assay is the best way to confirm this.

*In the text there is a big emphasis on the role of Jam-C in leukocyte transmigration. However, the data presented are regarding the dynamics of Jam-C in normal endothelial monolayers, scratch assays or TNF-treated endothelium. I recommend to either prove that CBL-mediated Jam-C protein trafficking is involved in transmigration of leukocytes across endothelium or to change the focus of the manuscript to better reflect the current results.*

We do understand the reviewers concerns about this aspect. The findings we have made are potentially relevant to all of JAM-Cs characterised functions. We feel it is therefore necessary to discuss the whereabouts of proteins associated with both leukocyte transmigration as well as permeability and transmigration. However we have attempted to shift the discussion to greater highlight the role of JAM-C in endothelial cell migration.

*I encourage the authors to improve the presentation of the figures by marking performed immuno-stainings in the figure itself rather than in the figure legends only.*

We have amended the figures to include such labelling.

*Figure 2B; how was the junctional area defined to be able to quantify whether Jam-C intensity levels?*

The fed antibody signal is lost relatively rapidly during antibody feeding and is therefore easy to quantify. We separated junctional labelling from vesicular by excluding any round objects and any objects which are less than 50 pixels using a cell profiler pipeline (see lines 634 to 637).

*What happens to VE-cadherin trafficking, which is also internalized through a similar mode of tubular endocytosis from remodeling junctions, once the lysines in Jam-C are mutated and preventing its proteosomal degradation. Does the trafficking of other receptors remain unaffected or is their a co-dependent mode of internalization and endocytosis?*

We could not see any evidence for a co-dependence of trafficking for NRP-1, 2 or VE-Cadherin with JAM-C. These experiments relied on knockdown of JAM-C and immunofluorescence analysis of the potential co-trafficked receptors. There is still scope for co-dependence of trafficking for other receptors such as the integrins, although this has yet to be characterised. There is also the potential for shared machinery for trafficking and we are currently investigating this.

1.	Park M, Raftery MJ, Thomas PS, Geczy CL, Bryant K, Tedla N. Leukocyte immunoglobulin-like receptor B4 regulates key signalling molecules involved in FcgammaRI-mediated clathrin-dependent endocytosis and phagocytosis. Sci Rep. 2016;6:35085. Epub 2016/10/12. doi: 10.1038/srep35085. PubMed PMID: 27725776; PubMed Central PMCID: PMCPMC5057125.

2.	Sulzer B, De Boer RJ, Perelson AS. Cross-linking reconsidered: binding and cross-linking fields and the cellular response. Biophys J. 1996;70(3):1154-68. Epub 1996/03/01. doi: 10.1016/S0006-3495(96)79676-5. PubMed PMID: 8785275; PubMed Central PMCID: PMCPMC1225045.

3.	Rhee HW, Zou P, Udeshi ND, Martell JD, Mootha VK, Carr SA, et al. Proteomic mapping of mitochondria in living cells via spatially restricted enzymatic tagging. Science. 2013;339(6125):1328-31. doi: 10.1126/science.1230593. PubMed PMID: 23371551; PubMed Central PMCID: PMCPMC3916822.

4.	Duval M, Bedard-Goulet S, Delisle C, Gratton JP. Vascular endothelial growth factor-dependent down-regulation of Flk-1/KDR involves Cbl-mediated ubiquitination. Consequences on nitric oxide production from endothelial cells. The Journal of biological chemistry. 2003;278(22):20091-7. Epub 2003/03/22. doi: 10.1074/jbc.M301410200. PubMed PMID: 12649282.
